# Supplementary figures and images for: Genome-wide identification of the MADS-box gene family in Avena sativa and its role in photoperiod-insensitive oat
Source: PeerJ. 2024 Jan 22;12:e16759. doi: 10.7717/peerj.16759 (PMC10809983; doi:10.7717/peerj.16759)

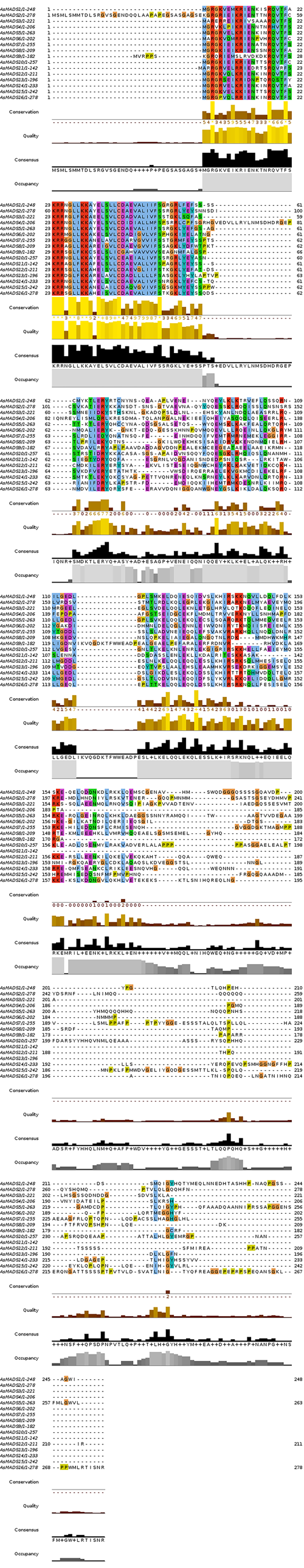

Supplement: Supplemental Information 1 [file peerj-12-16759-s001.png]
